# A Single-Institution Analysis of Thymic Carcinoma Treated with Multi-Modality Therapy

**Published:** 2017-11-22

**Authors:** Imran H Mohiuddin, Muhammad Furqan, Gerald Clamon, John Keech, Carryn Anderson, Mark C Smith, John M Buatti, Bryan G Allen

**Affiliations:** 1 Department of Radiation Oncology, University of Iowa, USA; 2 Department of Internal Medicine, Division of Hematology and Oncology, University of Iowa, USA; 3 Department of Surgery, Division of Thoracic Surgery, University of Iowa, USA

**Keywords:** Thymic carcinoma, Radiation therapy, Thymectomy

## Abstract

**Purpose:**

Review of our experience in treating thymic carcinoma patients using a combination of surgery, chemotherapy and radiation therapy.

**Methods:**

An institutional review of thymic carcinoma patients treated between 2007 and 2014 was performed analyzing clinical characteristics, treatment intent, surgical margin status, and radiation treatment dose. Survival curves were generated using the Kaplan-Meier method.

**Results:**

Nine individuals were treated for newly diagnosed thymic carcinoma. Three patients had unresectable disease at presentation; two of these were treated with definitive chemoradiation therapy while another received neoadjuvant chemotherapy. Seven subjects underwent surgical resection (one after neoadjuvant chemotherapy) with pathological staging ranging from IIa – IVb disease. Patients were planned for adjuvant radiotherapy followed by chemotherapy; however, one developed liver metastases prior to initiating radiotherapy and was therefore treated with palliative chemotherapy alone. A second patient was non-compliant with radiation treatments and was considered as treated with palliative chemotherapy alone. Of the seven patients who completed definitive treatment, median time to progression and overall survival has yet to be reached. Only one of these patients developed progressive disease 10 months after completing treatment and eventually succumbed to disease 41 months after completing definitive therapy. With a median follow up of 30 months, two year overall survival is 67% for all patients.

**Conclusion:**

Resection with an emphasis on best possible oncologic margins, followed by radiation and chemotherapy remains an effective treatment strategy for advanced stage thymic carcinoma. In patients who present with unresectable tumors, neoadjuvant chemotherapy or definitive chemoradiation therapy may also be considered as viable treatment strategies.

## Introduction

Thymic epithelial tumors collectively represent a rare set of anterior mediastinal tumors comprised of both thymomas and thymic carcinomas. Thymic carcinomas comprise 15% of all thymic tumors [1]. Thymic carcinomas and thymomas are often grouped together in chart-based studies despite the understanding that thymic carcinomas exhibit distinct clinical and pathologic features. Compared to thymomas, thymic carcinomas are not associated with myasthenia gravis or other autoimmune syndromes. Their clinical behavior is markedly more aggressive with respect to local invasiveness, nodal involvement, and distant metastasis, which results in worse clinical outcomes [2]. This behavior correlates with histologic features which include pronounced cellular atypia and poor resemblance to normal thymus glandular architecture. Thymic carcinomas are also unique in their expression of markers such as CD5 and CD117 [1].

The central importance of maximal surgical resection with an emphasis on best possible oncologic margins in the management of thymic carcinoma is well-appreciated [2]. Thymic carcinoma patients who have a R0 resection (complete excision with negative margins) have a 5 year overall survival of 60% while those with either a R1 (microscopically positive margins) or R2 (macroscopically positive margin) resection have 5 year overall survivals of 49%, and 32% respectively. Survival rate also greatly depends upon disease stage, as patients with Masaoka stages II, III, and IV a disease have a 5-year overall survival rates of 81%, 51%, and 24% respectively [3].

Unfortunately, advanced stage disease (≥ Masaoka stage III disease) at the time of diagnosis is common and often prevents negative surgical margins [4]. Consequently, the roles of neoadjuvant chemotherapy and/or adjuvant radiation therapy and chemotherapy merit further investigation. As described in the 2017 NCCN version 1 thymic carcinoma guidelines, thymic carcinoma patients with stage II–IV disease who undergo an R0 resection should be considered for adjuvant radiation therapy while patients who undergo R1 or R2 resection should be considered for adjuvant radiation and chemotherapy. Adjuvant radiation dose is dependent upon the extent of residual disease. Current NCCN guidelines suggest conventionally fractionated (1.8 Gy to 2 Gy per fraction) adjuvant radiation with total doses of 45 Gy to 50 Gy for a R0 resection, 54 Gy for a R1 resection, and ≥ 60 Gy for grossly positive margins [5–7]. It is currently recommended that patients who present with unresectable thymic carcinoma should receive neoadjuvant chemotherapy followed by re-evaluation for surgical resection. Patients who remain unresectable should receive definitive radiation and chemotherapy, while those who become resectable should receive surgery followed by the consideration of adjuvant radiation therapy and chemotherapy [3].

The rarity of thymic carcinomas precludes its prospective evaluation in a large series. Most thymic carcinoma reports are multi-decade retrospective series with many reports including both thymomas and thymic carcinomas. The extended period over which these cases occurred may also confound conclusions due to the the significant evolution in treatment strategies over the period of observation [3]. Consequently, there is poor consensus on optimal treatment strategies for thymic carcinoma. We attempt to address these issues by analyzing treatment outcomes from a recent patient cohort comprised solely of thymic carcinoma patients treated with definitive intent between the years of 2007 and 2014.

## Materials and Methods

### Patient eligibility

Patients with a pathological diagnosis of thymic carcinoma treated atour institution with planned defintiive radiation therapy between January 2007 and December 2014 were identified and their records were reviewed. Thymic carcinoma cases were characterized using Masaoka-Koga staging and International Thymic Malignancy Interest Group (ITMIG) conventions. Cases were analyzed in terms of clinical presentation, diagnostic workup, immunohistochemistry markers (cytokeratin, p63, CD5, and CD117), surgical resection margin status, pathology subtype, and overall clinical course. This study was approved by our Institutional Review Board.

### Radiation treatment planning

All patients underwent CT-based radiation treatment planning. Patient immobiliziation was obtained using either an arm board or custom-made positioning cushion. Patients received a contrast Computed Tomographic (CT) scan of 2 mm slice thickness. The Clinical Target Volume (CTV) included the thymic tumor bed and areas of concern for residual microscopic or residual disease based on discussion with the thoracic surgeons. The Planning Target Volume (PTV) included an additional 5 mm margin around the CTV. All patients received a 3D-conformal or IMRT based radiation. A curative intent radiation dose ranged from 45 Gy to 60 Gy, depending upon disease status at time of treatment planning, and was delivered using daily conventional fractionation Monday through Friday. Prior to radiation treatment delivery, an orthovoltage conebeam CT scan was acquired for localization.

### Chemotherapy planning

Chemotherapy treatments included neoadjuvant, adjuvant, and definitive treatment regimens. In the neoadjuvant setting, one patient received 3 cycles of ADOC (cisplatin 50 mg/m^2^ intravenous (IV) day 1, doxorubicin 40 mg/m^2^ IV day 1, vincristine 0.6 mg/m^2^ IV day 3, and cyclophosphamide 500 mg/m^2^ IV day 4) followed by surgical resection. In the adjuvant setting, patients received either carboplatin-paclitaxel (carboplatin AUC 5 and paclitaxel 200 mg/m^2^ IV day 1 of every 3 weeks) or ADOC chemotherapy following completion of radiation therapy. In the two cases of definitive concurrent radiation and chemotherapy in the setting of unresectable disease, one patient received concurrent carboplatin-paclitaxel and the second who had synchronous multiple myeloma received bortezomib and dexamethasone. Two patients receiving palliative chemotherapy received either carboplatin-paclitaxel (carboplatin AUC 5 and paclitaxel 200 g mg/m^2^ IV day 1 of every 3 weeks) or etoposide-cisplatin (etoposide 100 mg/m^2^ and cisplatin 60 mg/m^2^ day 1 of every 3 weeks).

### Response evaluation

Patients were assessed for treatment-related toxicity and disease progression or recurrence at scheduled follow-up visits generally performed every three months with imaging following treatment and with decreased frequency over time. Toxicities were defined and graded using the Common Terminology Criteria for Adverse Events v.4.0 (CTCAE). Time to progression was defined as the number of months elapsed from obtaining tumor histology to tumor recurrence based on CT-based imagingas defined by the absence of progression at the treatment site, per RECIST 1.1 criteria or development of metastatic disease. Overall survival was defined as the number of months elapsed from obtaining tumor histology to death from any cause. Survival curves were generated by the Kaplan-Meier method.

## Results

### Patient characteristics

Between January 01, 2007 and December 31, 2014, nine individuals were identified who met the study inclusion criteria. The patient characteristics are listed in Table 1. Identified patients included six men and three women with histologies classifiedas either: poorly-differentiated thymic carcinoma, thymic carcinoma not otherwise specified, or squamous-cell-type thymic carcinoma. Seven thymic carcinoma patients underwent extended thymectomy with resection of any involved tissue or structures through a transsternal approach (three with a R0 resection, one with aR1 resection, and three with a R2 resection) with final pathologic Masaoka staging ranging from IIa to IVb disease. A single patient with stage IVb disease underwent neoadjuvant ADOC-based chemotherapy due to unresectable disease at presentation with subsequent R0 resection (Table 1 patient 1). Five patients were found to have poorly differentiated thymic carcinomas, three with thymic carcinoma squamous cell carcinoma type, and one with thymic carcinoma not otherwise specified. Two patients were found to have other wise unresectable disease at presentation and were treated with definitive chemoradiotherapy with one patient receiving concurrent weekly carboplatin and paclitaxel and another patient receiving concurrent bortezomib and dexamethasone for treatment along with synchronous diagnosis of multiple myeloma.

### Adjuvant treatment regimens

Postresection, patients were planned to receive conformal adjuvant radiotherapy to the thymic tumor bed to a dose of 45 Gy to 54 Gy in conventional 1.8 Gy to 2.0 Gy fractions). Patients with residual disease or those with unresectable disease received further radiation treatments up to a total dose up to 66.6 Gy utilizing a shrinking volume/cone-down approach. However, one patient with R2-surgical margins had poor compliance with treatment sessions and pre-maturely stopped therapy after receiving 30 Gy. He went on to receive palliative chemotherapy with carboplatin-paclitaxol. Another patient with R2-surgical margins rapidly developed liver metastases prior to starting radiation therapy and was subsequently treated with palliative chemotherapy with cisplatin and etoposide. In general, radiation therapy was followed by 4–6 weeks of chemotherapy with either ADOC or carboplatin-paclitaxel regimens.

### Patient outcomes

Overall, patients tolerated adjuvant radiation therapy well with development of only mild (grade 1) erythema in four patients which shortly resolved after completion of radiation therapy. There were no reported or observed long term radiation-related sequelae.

**Figure 1:**
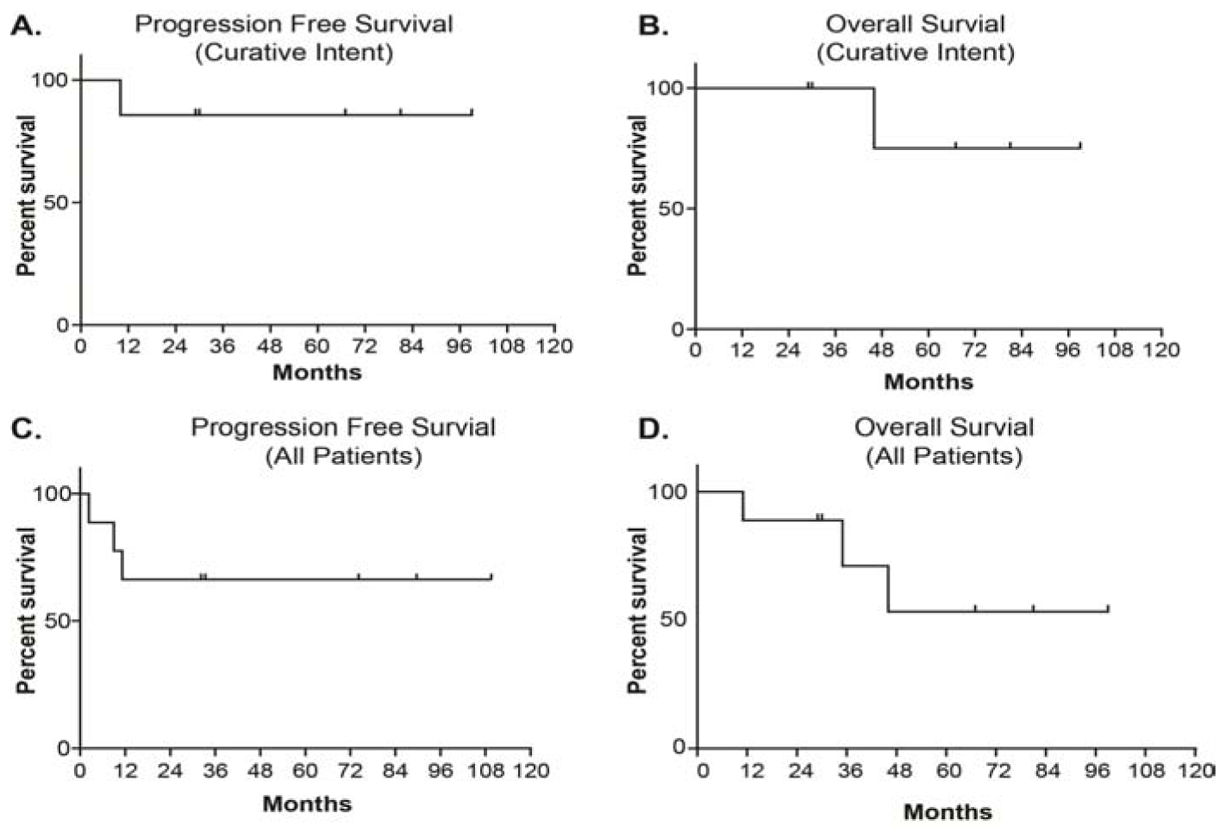
For curative intent treated thymic carcinoma patients, (A) progression free survival was defined as the number of months elapsed from obtaining tumor histology to tumor recurrence, (B) Overall survival was defined as the number of months elapsed from obtaining tumor histology to death from any cause. Graphs C and D similarly represent progression-free and overall survival, inclusive of all patients (curative and palliative intent treated patients).

In the context of clinical outcomes, the patient who received neoadjuvant ADOC chemotherapy followed by an R0 resection had disease progression 10 months after therapy. This individual demonstrated a 41 month overall survival, and ultimately succumbed to metastatic brain disease (Table 1 patient 1). The two patients who received definitive concurrent chemoradiation therapy are alive and well with no evidence of disease progression 67 and 77 months after completing therapy respectively (Table 1 patients 5 and 7). Two patients underwent R0 surgical resection followed by adjuvant radiation therapy and chemotherapy are alive and well with no evidence of disease progression at 29 and 30 months after completing therapy respectively (Table 1 patients 2 and 3). One patient underwent an R1 resection followed by adjuvant radiation therapy and chemotherapy experienced a myocardial infarction 18 months after completing therapy (Table 1 patient 4); this individual had significant cardiac risk factors prior to initiating thymic carcinoma therapy. One patient underwent an R2 resection followed by adjuvant radiation and chemotherapy and is alive and well 76 months after completing therapy without evidence of disease progression (Table 1 patient 6). The patient who was found to have metastatic disease prior to beginning radiation therapy lived for 11 months following surgical resection (Table 1 patient 8). The patient who stopped radiation therapy prematurely after receiving 30 Gy also lived for 11 months following surgical resection (Table 1 patient 9). With a median follow up of 30 months, two year overall survival is 67% in all nine patients. Including all patients, 83% were alive at one year, 67% were alive at two years, and 33% of patients have survived for greater than five years (Figure 1 and Table 1). Currently five patients remain alive with no evidence of disease progression.

## Discussion

Thymic carcinomas are rare tumors of the mediastinum that comprise 15% of all thymic tumors [3]. Compared to thymomas, thymic carcinomas are reported to have greater tendency to invade surrounding structures, higher metastatic potential and worse outcomes [8]. Whereas many other studies have either combined thymoma and thymic carcinoma for a collective study of all thymic epithelial tumors [9], or inspected a large cohort of thymic carcinoma patients over a long (20+ year) period during which treatment has evolved [3], our study is unique in comprising solely thymic carcinoma patients treated in a modern era (year 2000 and onward) of image-based conformal radiation treatment delivery.

The mainstay of treatment for thymic carcinoma patients without evidence of extra thoracic metastases is surgical resection with an emphasis on optimal oncologic margins [10–12]. All thymic carcinoma patients who have Masaoka stage II disease or greater should be considered for adjuvant radiation therapy with total delivered dose dependent upon the completeness of surgical resection. Patients who have unresectable disease or residual gross disease following resection should receive a total radiation dose ≥ 60 Gy with chemotherapy [13,14]. For patients with residual microscopic disease, the tumor bed should receive a total dose of 54 Gy, while in those patients with negative or close margins; a dose of 45 Gy to 50 Gy should be administered [5–7].

Though patterns of care analyses utilizing national cancer databases suggest that the utilization of radiation treatment in the management of thymic carcinoma continues to increase, there are conflicting reports as to whether adjuvant radiotherapy confers any survival benefit [15,16]. Whereas an apparent majority of the literature agrees on the importance of maximal resection and chemotherapy, large multivariate analyses have failed to observe utilization of radiotherapy as an independent prognosticator for survival [17]. This apparent conflict may be resolved in the context of others’ findings suggesting a benefit observed only in certain histological subtypes or in those who also received chemotherapy [18].

Survival rates vary depending on stage, resectability and completeness of resection. Patients with Masaoka stage II, III, IV a disease have a 5-year overall survival of 81%, 51% and 24% respectively [3]. In our study, two patients had stage II disease, with one patient dying from a myocardial infarction 18 months after surgical resection and one patient doing well with no evidence of disease recurrence 29 months after surgery. Five patients had stage III disease, with four patients having no evidence of disease recurrence (30 months, 67 months, 77 months, and 76 months) after surgical resection and one patient being planned for but not receiving any radiation treatments due to discovery of metastatic disease and hence receiving palliative chemotherapy alone. This individual lived for 11 months following surgical resection. Two patients had stage IV disease with one patient developing a local recurrence in the mediastinum 10 months following completion of definitive chemoradiotherapy with an overall survival of 46 months. The other stage IV patient did not complete the planned radiation treatment and received palliative chemotherapy dying 11 months after surgical resection.

Our results support the importance of up-front maximal resection for optimal outcome [19], with disease recurrence and progression mainly occurring in patients with incomplete (R2) oncologic resections. Patients with a R0 resection have a 5-year overall survival of 60%, R1 of 49%, and R2 of 32%, respectively [3]. In our series, three patients had an R0 surgical resection with one patient developing recurrence 10 months after completion of definitive therapy. One patient had a R1 surgical resection that had no evidence of disease recurrence 18 months after completion of definitive therapy. Three patients had R2 surgical resections with only one patient demonstrating no evidence of disease progression 76 months after therapy.

If the likelihood of residual disease is high after surgery, or if disease is unresectable, neoadjuvant chemotherapy should be considered as 1 of our patients who presented with initially unresectable disease received neoadjuvant chemotherapy and achieved a R0 resection. In our experience, treatment failure consistently occurred outside of radiation fields, which highlights the importance of adjuvant RT. In this framework, a vital role for adjuvant radiation and chemotherapy is demonstrated with excellent and sustained time to progression and overall survival of patients who presented with locally advanced thymic caricinoma.

## Conclusions

Maximal safe surgical resection with an emphasis on the best possible oncologic margins in those deemed surgically resectable, followed by adjuvant radiotherapy and chemotherapy, and remains an effective treatment strategy for locally advanced stage thymic carcinoma. In patients who present with initially unresectable disease, neoadjuvant chemotherapy may be a viable approach for local control. Alternatively, definitive radiotherapy and chemotherapy with dose escalation to ≥ 60 Gy may also be considered as an effective treatment option for those unable to have resection.

## Figures and Tables

**Table 1:** Thymic carcinoma patient demographics and characteristics are summarized as gender (male *vs.* female), age at diagnosis (age at dx), ethnicity, histology, immunohistochemistry (IHC), and Masaoka stage at diagnosis. Treatment intent and sequencing are indicated. Oncologic margins listed as gross total resection (R0), residual microscopic disease (R1), and gross disease remaining (R2). Total radiation dose is listed in units of grays. Time to progression and overall survival for patients who have had confirmed recurrence and died are listed in months.

Patient	Sex	Age at Dx	Histology	IHC	Stage	Treatment Sequence	Resection	Radiation Dose (Gy)	Time to Progression (months)	Overall survival (months)
1	M	62	PD	CD5+, CD117+	IVb	chemo → surgery → RT	R0	60	10	46
2	M	70	NOS	CD5+, CD117+	III	surgery → RT → chemo	R0	54	30*	30*
3	F	53	SC	CD5+, CD117+	IIb	surgery → RT → chemo	R0	45	29*	29*
4	M	66	PD	CD5−, CD117−	IIa	surgery → RT → chemo	R1	50.4	18	18
5	M	70	SC	-	III	Concurrent chemo-RT for MM	Inoperable	66.6	67*	67*
6	F	47	SC	-	III	surgery → RT → chemo	R2	59.4	77*	77*
7	M	48	PD	CD5−, CD117+	III	Concurrent chemo-RT	Inoperable	63.2	76*	76*
8	M	19	PD	CD5+, CD117−	IVb	Surgery → palliative chemo	R2	30	0	11
9	F	73	PD	CD5−, CD117−	III	Surgery → palliative chemo	R2	None	6	11

“MM” indicates a patient with synchronous multiple myeloma; “*” indicates patients who are alive with no evidence of disease progression; “PD” is poorly differentiated thymic carcinoma; “NOS” is thymic carcinoma not otherwise specified; “SC” is thymic carcinoma, squamous cell carcinoma type.
